# Suspected liraglutide (glucagon-like peptide-1 receptor agonist)-induced hyperthyroidism: A case report

**DOI:** 10.51866/cr.886

**Published:** 2025-08-07

**Authors:** Siti Nur Syakinah Md Sabudin, Lili Husniati Yaacob, Muhammad Rahmat Che Omar

**Affiliations:** 1 MBBS, MMed (Family Medicine), Department of Family Medicine, School of Medical Sciences, Health Campus, Universiti Sains Malaysia, Kubang Kerian, Kelantan, Malaysia. E-mail: husniati@usm.my; 2 MBBS, MMed (Family Medicine), Medical and Basic Dental Sciences Unit, School of Dental Sciences, Health Campus, Universiti Sains Malaysia, Kubang Kerian, Kelantan, Malaysia.; 3 MBBS, Pejabat Kesihatan Daerah Bachok, Bachok, Kelantan, Malaysia.

**Keywords:** Liraglutide, Hyperthyroidism, Adverse drug reaction

## Abstract

Liraglutide, a glucagon-like peptide-1 receptor agonist (GLP-1 RA), is widely used for weight management and glycaemic control. While generally well-tolerated, various adverse effects have been reported. Thyroid dysfunction, particularly hyperthyroidism, is rare. This report describes the case of a 34-year-old woman with new-onset hyperthyroidism following liraglutide use for weight loss. The patient presented with worsening palpitations and insomnia after 3 weeks of liraglutide use. She had no prior thyroid disease or significant family history. Examination revealed sinus tachycardia with no goitre or tremors. Laboratory findings confirmed hyperthyroidism with a suppressed TSH level (0.03 mlU/L) and an elevated free T4 level (20.8 pmol/L). Thyrotropin receptor antibody was negative, ruling out Graves’ disease. Liraglutide was discontinued, and the patient was treated with carbimazole and propranolol, resulting in symptom resolution and normalisation of thyroid function within weeks. Liraglutide-induced thyroid dysfunction is poorly understood. Possible mechanisms include GLP-1 receptor activity in thyroid tissue, disruption of the hypothalamic-pituitary-thyroid axis or inflammation of thyroid follicular cells. This case demonstrates a strong temporal association between liraglutide use and hyperthyroidism, which resolved upon drug discontinuation. Although thyroid dysfunction with GLP-1 RAs has been noted in preclinical studies, human data remain limited. This case highlights a rare adverse effect of liraglutide and underscores the need for vigilance when monitoring patients on GLP-1 RAs. Monitoring of thyroid function should be considered in symptomatic patients. Further research is warranted to better understand the underlying mechanisms and prevalence of GLP-1 RA-induced thyroid abnormalities.

## Introduction

Adverse drug reactions remain a critical concern in modern medicine, particularly with the increasing use of novel therapeutic agents. Liraglutide, a glucagon-like peptide-1 receptor agonist (GLP-1 RA), has gained widespread popularity for its efficacy in managing type 2 diabetes and obesity. While liraglutide is generally well-tolerated, potential adverse effects, including gastrointestinal disturbances, pancreatitis and thyroid-related changes, have been documented in clinical trials and post-marketing surveillance.^1^

Thyroid dysfunction associated with liraglutide use is rare, and most reported cases involve benign thyroid nodules or C-cell hyperplasia observed in preclinical studies.^2^ However, hyperthyroidism is an uncommon and poorly understood adverse effect. This report describes the case of a 34-year-old woman presenting with new-onset hyperthyroidism following liraglutide use for weight loss, highlighting the importance of vigilance and monitoring for rare but significant adverse effects.

## Case presentation

A 34-year-old woman with no prior medical illness presented with worsening palpitations and insomnia for 1 week without chest pain, shortness of breath, irritability, excessive sweating, neck swelling or pain, fever and recent infection. She was para 2+2, with her last childbirth 2 years ago. She had no family history of thyroid disease. Notably, her symptoms began 3 weeks after initiating liraglutide, which she obtained from a private clinic for weight management due to a body mass index (BMI) of 24 kg/m^2^. Liraglutide was started at 0.6 mg daily and titrated every 5 days to 1.2 mg, 1.8 mg and eventually 2.4 mg at the time of presentation. She was not on any other medications, weight loss products or supplements.

Examination revealed sinus tachycardia (pulse rate: 134 bpm) and fine tremors. Other vital signs were normal, and there was no exophthalmos, goitre, thyroid tenderness, palpable thyroid nodules or other signs of hyperthyroidism. Other system examinations demonstrated unremarkable findings. Thyroid function test showed a suppressed TSH level (0.03 mlU/L; normal range: 0.40-4.00 mlU/L) and an elevated free T4 level (20.8 pmol/L; normal range: 7.86-14.41 pmol/L), with normal thyrotropin receptor antibody (TRAb) level (0.37 IU/L; normal range: 0.00-1.75 IU/L) and complete blood count.

Liraglutide was immediately discontinued, and she was started on carbimazole 10 mg daily and propranolol 20 mg TDS. Within 2 weeks, her symptoms improved significantly, and repeat thyroid function tests showed normal TSH (0.98 mlU/L) and free T4 levels (11 pmol/L). Propranolol was stopped, and carbimazole was tapered to 5 mg daily and then discontinued after a month when thyroid function remained normal (TSH level: 2.85 mlU/L, free T4 level: 13.5 pmol/L). Repeat thyroid function test a month after cessation of carbimazole showed that the TSH and free T4 levels remained normal.

The patient remained asymptomatic, and her thyroid function remained normal, with follow-up every 2 months for 6 months and then every 6 months thereafter. Thyroid ultrasound was subsequently performed at follow-up, which showed normal findings. The diagnosis of thyroiditis was less likely due to the absence of thyroid pain, tenderness, recent infections, concomitant medication use, goitre, abnormal complete blood count and any episodes of hypothyroidism during follow-up. The normal TRAb level decreased the likelihood of Graves’ disease.

Given the close temporal relationship, absence of an alternative cause and resolution after stopping the drug, liraglutide-induced hyperthyroidism was strongly suspected.

## Discussion

Liraglutide is a GLP-1 RA commonly prescribed for weight management and glycaemic control. While preclinical studies have raised concerns about its association with thyroid C-cell hyperplasia and medullary thyroid carcinoma,^2^ its role in thyroid hormone dysregulation remains poorly understood. Potential mechanisms of hyperthyroidism include GLP-1 receptor activity in thyroid tissue in which stimulation of GLP-1 receptors expressed on thyroid cells may influence thyroid hormone synthesis or secretion, leading to transient hyperthyroidism.^4,5^ Thyroid function is disrupted by directly altering the hypothalamic-pituitary-thyroid axis or inducing inflammation in thyroid follicular cells, resulting in thyroiditis. Additionally, GLP-1 RAs could theoretically interact with the immune system, although there is limited evidence linking liraglutide to autoimmune thyroid disorders.^4-7^

Although liraglutide-induced hyperthyroidism is not widely reported, thyroid dysfunction has been observed in the context of GLP-1 RA use.^8,9^ Preclinical studies in rodents have highlighted a potential link between GLP-1 RAs and thyroid C-cell pathology, although these findings have not been conclusively replicated in humans.^2^ It is important to note the possible species-specific effects of GLP-1 RAs on thyroid hormones; therefore, caution should be exercised when extrapolating the same result to humans.

A similar case showing the possible impact of GLP-1 RAs on thyroid hormones was recently reported. The report illustrated the case of a post-thyroidectomy patient on levothyroxine with reduced TSH levels following subcutaneous semaglutide administration.^3^ In this case, the patient was on levothyroxine 200 mcg daily for the past 5 years and had normal thyroid hormone levels before the presentation. After the initiation and titration of semaglutide, the TSH level was suppressed, but the free T4 level remained within the normal range, and the patient was asymptomatic. Her levothyroxine dosage was lowered by 25% to 150 mcg orally daily to restore her thyroid hormone level to normal in response to the decline in her TSH level. This case showed the possibility that GLP-1 RAs could affect the thyroid hormone levels.

In our case report, the temporal association between the initiation of liraglutide and the onset of hyperthyroid symptoms, along with the resolution of symptoms and normalisation of thyroid function upon cessation of liraglutide, strongly supports the hypothesis of a causal relationship. Key considerations for drug-induced thyroid dysfunction included the absence of other medications, sign of infections or iodine exposure and a normal thyroid function a year prior, ruling out preexisting thyroid dysfunction. However, the causal relationship could be further strengthened if the symptoms recur with rechallenge with the same drug, which was not conducted in this case.

The management in our case included immediate discontinuation of liraglutide, initiation of carbimazole to suppress thyroid hormone synthesis and administration of propranolol for symptomatic relief of tachycardia. The patient’s symptoms improved rapidly, and her thyroid function normalised within weeks. The antithyroid medication was tapered and eventually discontinued, with sustained euthyroid status at follow-up.

Given that the patient took liraglutide for weight loss, it was important to address longterm weight management following the cessation of the medication. She was advised on the implementation of sustainable lifestyle modifications, including adopting a balanced diet and engaging in regular physical activity. She was advised on a suitable exercise regimen, and referral was made to a dietitian for individualised nutritional counselling. Pharmacological therapy for obesity was not initiated. According to the Malaysian Clinical Practice Guidelines on the Management of Obesity 2023, pharmacotherapy is recommended for individuals with a BMI of ≥30 kg/m^2^ or ≥27.0 kg/m^2^ with the presence of obesity-related comorbidities.^8^ As the patient’s BMI was 24 kg/m^2^ and she had no associated comorbid conditions, she did not meet the current indication for obesity pharmacotherapy. Regular follow-up was arranged to monitor both her weight status and thyroid function.

The evidence regarding the effects of GLP-1 on thyroid function is still limited and controversial. Other studies have suggested that the activation of GLP-1 RAs does not influence the thyroid hormone levels.^9-11^ While this case strongly implicates liraglutide in the development of hyperthyroidism, it remains a single case report, and causality cannot be definitively established. The association between liraglutide and hyperthyroidism has not been firmly established. Therefore, a prompt and thorough medical evaluation is essential to exclude other potential causes before attributing the condition to liraglutide-induced hyperthyroidism. Further research is needed to investigate the prevalence of thyroid dysfunction among liraglutide users, identify risk factors that predispose individuals to thyroid dysfunction and explore the underlying mechanisms of GLP-1 RA-induced thyroid abnormalities. Health practitioners should practise vigilance for potential thyroid hormone dysregulation in patients prescribed with GLP-1 RAs until further evidence can shed light on the issue.

## Conclusion

This case highlights a potential rare adverse effect of liraglutide on thyroid hormones, emphasising the need for vigilance in monitoring for thyroid symptoms in patients receiving GLP-1 RAs. Clinicians should be aware of this potential complication, particularly as the use of liraglutide for weight management increases. Early recognition and management can prevent complications and ensure a favourable outcome.
